# Central obesity as assessed by conicity index and a-body shape index associates with cardiovascular risk factors and mortality in kidney failure patients

**DOI:** 10.3389/fnut.2023.1035343

**Published:** 2023-03-01

**Authors:** Kakei Ryu, Mohamed E. Suliman, Abdul Rashid Qureshi, Zhimin Chen, Carla Maria Avesani, Torkel B. Brismar, Jonaz Ripsweden, Peter Barany, Olof Heimbürger, Peter Stenvinkel, Bengt Lindholm

**Affiliations:** ^1^Division of Renal Medicine and Baxter Novum, Department of Clinical Sciences, Intervention and Technology, Karolinska Institutet, Stockholm, Sweden; ^2^Clinical Research Institute for Clinical Pharmacology and Therapeutics, Showa University, Tokyo, Japan; ^3^Kidney Disease Center, 1st Affiliated Hospital College of Medicine, Zhejiang University, Hangzhou, China; ^4^Unit of Radiology, Department of Clinical Sciences, Intervention and Technology, Karolinska Institutet, Stockholm, Sweden; ^5^Department of Radiology, Medical Diagnostics Karolinska, Karolinska University Hospital, Stockholm, Sweden

**Keywords:** central obesity, waist circumference, a body shape index, conicity index, cardiovascular risk, mortality, kidney failure patients

## Abstract

**Background:**

Anthropometric indices of central obesity, waist circumference (WC), conicity index (CI), and a-body shape index (ABSI), are prognostic indicators of cardiovascular (CV) risk. The association of CI and ABSI with other CV risk indices, markers of nutritional status and inflammation, and clinical outcomes in chronic kidney disease (CKD) stage 5 (CKD5) patients was investigated.

**Methods:**

In a cross-sectional study with longitudinal follow up of 203 clinically stable patients with CKD5 (median age 56 years; 68% males, 17% diabetics, 22% with CV disease, and 39% malnourished), we investigated CI and ABSI and their associations with atherogenic index of plasma (AIP), Framingham CV risk score (FRS), Agatston scoring of coronary artery calcium (CAC) and aortic valve calcium (AVC), handgrip strength (HGS), high sensitivity C-reactive protein (hsCRP) and interleukin-6 (IL-6). CV events (CVE) and all-cause mortality during up to 10-years follow up were analyzed by multivariate survival analysis of restricted mean survival time (RMST).

**Results:**

Chronic kidney disease patients with middle and highest CI and ABSI tertiles (indicating greater abdominal fat deposition), compared to those with the lowest CI and ABSI tertiles, tended to be older, more often men and diabetic, had significantly higher levels of hsCRP, IL-6, AIP, FRS, CAC and AVC scores. CI and ABSI were positively correlated with CAC, FRS, AIP, hsCRP and IL-6. Both CI and ABSI were negatively correlated with HGS. In age-weighted survival analysis, higher CI and ABSI were associated with higher risk of CVE (Wald test = 4.92, *p* = 0.027; Wald test = 4.95, *p* = 0.026, respectively) and all-cause mortality (Wald test = 5.24, *p* = 0.022; Wald test = 5.19, *p* = 0.023, respectively). In RMST analysis, low vs. high and middle tertiles of CI and ABSI associated with prolonged CVE-free time and death-free time, and these differences between groups increased over time.

**Conclusion:**

Abdominal fat deposit indices, CI and ABSI, predicted CV outcomes and all-cause mortality, and were significantly associated with the inflammatory status in CKD patients.

## 1. Introduction

Obesity is a growing public health problem affecting a substantial and rapidly increasing proportion of the population across different ages and racial/ethnic groups (1). Adipose tissue is a metabolically active endocrine organ (2) and an increase in fat mass has a significant impact on metabolic risk factors (3). Obesity is accompanied by a wide range of complications, including kidney damage (1, 4) and the prevalence of obesity is rising among patients with chronic kidney disease (CKD) (5). Obesity may cause kidney damage because of closely linked comorbidities, such as type 2 diabetes, hypertension, dyslipidemia, and accelerated atherosclerosis, and through direct effects on the kidneys mediated through hemodynamic and hormonal influences, insulin resistance, low-grade inflammation, adipokines, oxidative stress, protein glycation, and endothelial dysfunction (6, 7).

Fat mass and its distribution can be assessed by imaging techniques such as computed tomography and magnetic resonance as well as by ultrasound and dual energy X-ray absorptiometry (DXA). However, the use of these methods in clinical practice and in research may be limited by restricted availability of expensive instruments, high costs of maintenance, and need of expert operators (8–10).

An alternative option to assess adiposity is to use anthropometric measurements, which are cost-effective, non-invasive, readily available, and affordable methods to assess obesity, but not always reliable for precise diagnose (8–10). Historically, the most common method for defining obesity is body mass index (BMI), which can be considered as a crude marker of general obesity (11). However, BMI does not differentiate fat mass from lean body mass, cannot precisely diagnose adiposity (12) and it does not account for body fat distribution in different body regions (11, 12). Moreover, BMI may be influenced by volume overload, which is common in CKD. Therefore, BMI does not convince as a reliable measure of body fat content in CKD patients (13).

In recent years, it has become clear that different regional adipose tissue locations have different metabolic implications and matters more than total adipose tissue mass (3) and the focus has been directed to the importance of the regional distribution of body fat, specifically central obesity, which cannot be assessed by BMI. Waist circumference (WC), a measure of central obesity, has been suggested as a better substitute for BMI (14) as abdominal obesity is more closely related to morbidity and mortality than BMI (15). In both renal and non-renal populations, abdominal fat has been more significantly associated with increased mortality than total or peripheral fat (16–20), suggesting that the location of adipose tissue, rather than the total fat mass, is a main determinant of metabolic and inflammatory consequences of obesity in CKD (21).

Specific anthropometric indices of central obesity, which are derived from WC, namely conicity index (CI) and a-body shape index (ABSI), have been suggested as possibly better prognostic indicators than BMI. The CI is an index of central obesity using WC, height, and weight to assess fat distribution (22) while ABSI is an index based on WC that is nearly independent of height, weight, and BMI (23).

The current study was undertaken to investigate the association of CI and ABSI with cardiovascular risk indices, nutritional and inflammatory markers, and the clinical outcome in CKD stage 5 patients.

## 2. Patient and methods

In this cross-sectional observational study with analyses of longitudinal data, we investigated CI and ABSI in 203 clinically stable patients with CKD stage 5 (CKD5) including 104 non-dialyzed (CKD5-ND) patients close to the initiation of dialysis therapy and 99 dialyzed (CKD5-D) patients treated by peritoneal dialysis (PD, *n* = 67) or hemodialysis (HD, *n* = 32). The patients were recruited from three cohort studies, Kärltx (24) including 80 patients, MIA (25) including 73 patients, and MIMICK2 (26) including 50 patients, performed at Department of Renal Medicine, Karolinska University Hospital, Stockholm, Sweden. Inclusion criteria were CKD5, age > 18 years, and available data on WC and coronary artery calcification (CAC) score. Exclusion criteria were signs of overt clinical infection, unwillingness to participate and no measurements of WC or CAC. The study was conducted in adherence to the Declaration of Helsinki and authorized by the Swedish Ethical Review Authority. A written informed consent was gained from each patient.

*Waist circumference* (WC) was measured at a level midway between the inferior margin of the last rib and the uppermost lateral iliac crest in standing position. *Body mass index* (BMI) was calculated as the subject’s body weight in kilograms divided by the square of the subject’s height in meters (kg/m^2^). *Conicity index* (CI) was calculated according to the equation defined by Valdez et al. (22), as follows:


$$\mathrm{CI}=\frac{\mathrm{waist circumference}\;\left(m\right)}{0.109\times \sqrt{\frac{\mathrm{weight}\;\left(\mathrm{kg}\right)}{\mathrm{height}\;\left(m\right)}}}.$$

*A Body Shape Index* (ABSI) was calculated using the following formula (23):


$$\mathrm{ABSI}=\frac{\mathrm{waist circumference}\;\left(m\right)}{{\mathrm{BMI}}^{\frac{2}{3}}\times \mathrm{height}\;{\left(m\right)}^{\frac{1}{2}}}$$

*Atherogenic index of plasma* (AIP) was calculated as the logarithmically transformed ratio of serum triglycerides (TG) to high-density lipoprotein cholesterol (HDL-C) as in this formula (27):


$$\mathrm{AIP}=\log\frac{\mathrm{TG}}{\mathrm{HDL}-C}.$$

*Framingham cardiovascular disease* (CVD) *risk score* (FRS), an estimate of 10-year risk of developing CVD, was calculated from age and sex stratified tables with scores for diabetes, systolic blood pressure (SBP), anti-hypertensive medication, total cholesterol, HDL-C and smoking habit (28). *Coronary artery calcium* (CAC) *score* and *aortic valve calcium* (AVC) *score* were measured using the scoring system described by Agatston et al. (29) as briefly defined elsewhere (30).

*Subjective global assessment* (SGA) of nutritional status was evaluated using questionnaire and physical examination (31). Based on this assessment, each patient received a nutritional status score: (1) normal nutritional status, (2) mild malnutrition, (3) moderate malnutrition or (4) severe malnutrition. In this study, malnutrition was defined as an SGA score > 1.

*Handgrip strength* (HGS) was determined in both hands by using a Harpenden Handgrip Dynamometer (Yamar, Jackson, MI, United States). Each measurement was repeated three times for each arm, and the highest value for each arm listed. For HD patients, we measured the right arm handgrip strength, because fistulas were usually located in the left arm.

*Circulating biomarkers* reflecting cardiovascular risk, nutritional status, and inflammation: Plasma and serum were separated and kept frozen at −70°C if not analyzed immediately. High-sensitivity C-reactive protein (hsCRP) was measured by the nephelometry method. Interleukin-6 (IL-6) was measured with ELISA commercial kits (Roche Diagnostics GmbH, Penzberg, Germany). Serum cholesterol, HDL-C and TG were analyzed by standard enzymatic procedures (Boehringer Mannheim, Mannheim, Germany). Low-density lipoprotein (LDL) cholesterol was calculated according to the Friedewald et al. (32) formula. The remaining biochemical analyses were done using routine methods at Karolinska University Hospital at Huddinge.

*Primary outcome* was cardiovascular events (CVE) and all-cause mortality. Follow-up time was up to 10 years (median 5.7 years, interquartile range, IQR, 2.9–8.9 years). Clinical and outcome data was retrieved from patient records. CVE was defined as occurrence after inclusion of one or more of the following events: myocardial infarct (non-ST-elevation myocardial infarction, NSTEMI, acute myocardial infarction, AMI), onset of ischemic heart disease requiring percutaneous coronary intervention (PCI) and coronary artery bypass grafting (CABG), *transient ischemic attack,* stroke, peripheral vascular ischemia, or severe aortic valve stenosis requiring surgery.

### 2.1. Statistical analyses

Data are expressed as median (IQR, interquartile range), median (95% confidence interval) or number (percentage), as appropriate. Statistical significance was set at the level of *p* < 0.05. Comparisons between two groups were assessed with the non-parametric Wilcoxon test for continuous variables and Chi-square test for nominal variables. Non-parametric Spearman rank correlation analysis was used to determine associations between variables and a multivariate linear regression analysis to determine the independent associated variables with CI and ABSI. Survival analyses and CVE were made with multivariate survival curve, using the lowest tertile of the CI or ABSI as reference in age-weighted analysis.

We analyzed event-free time of CV events and all-cause mortality using multivariate analysis of *restricted mean survival time (RMST)* which is a novel alternative to Cox proportional hazards model that can be applied also when the proportional hazards assumption is not fulfilled (33). The average length of event-free time until CVE or death occurred was calculated using RMST from baseline to a particular time point during 10 years of follow up. The time difference of RMST (ΔRMST) representing the difference between low tertile versus high and middle tertiles of CI or ABSI of mean event-free time was calculated by subtracting the RMST for the low tertile of CI or ABSI from the RMST for the high and middle tertile.

Statistical analyses were performed using Statistical Software Stata 17.0 (Stata Corporation, College Station, TX, USA), Statistical Analysis Systems (SAS) version 9.4 level 1 7 M (SAS Campus Drive, Cary, NC, United States).

## 3. Results

Conicity index and ABSI were measured in 203 CKD patients with median age 56 years; 68% of the patients were males, 17% were diabetic, 22% had CVD, and 39% were malnourished (Tables 1, 2).

**Table 1:** Baseline clinical and biochemical characteristics of 203 chronic kidney disease stage 5 (CKD5) patients according to tertiles of conicity index (CI).

	All	Low tertile	Mid + high tertiles	Value of *p*
	*N* = 203	*N* = 67	*N* = 136	
CI	1.3 (1.3–1.4)	1.2 (1.2–1.3)	1.4 (1.3–1.4)	<0.001
Age, years	56.0 (43.5–66.8)	45.0 (31.0–58.0)	61.0 (50.0–68.5)	<0.001
Male sex, *n* (%)	138 (68.0%)	36 (53.7%)	102 (75.0%)	<0.01
Diabetes mellitus, *n* (%)	34 (17.1%)	6 (9.1%)	28 (21.1%)	<0.05
CVD, *n* (%)	43 (21.5%)	12 (18.2%)	31 (23.1%)	ns
FRS%	13.6 (5.6–27.2)	6.3 (2.3–14.8)	17.6 (8.1–29.7)	<0.001
Systolic BP, mmHg	143 (130–153)	147 (131–156)	140 (129–152)	ns
Diastolic BP, mmHg	86 (76–93)	87 (78–96)	85 (76–92)	ns
Malnutrition [SGA(*n* = 198)]	77 (38.9%)	32 (47.8%)	45 (34.4%)	ns
Height, cm	173 (165–180)	171 (162–179)	174 (167–181)	ns
Weight, kg	74 (65.0–84.3)	65 (56.5–79.4)	76 (69.1–86.3)	<0.001
BMI, kg/m^2^	24.6 (22.2–27.5)	22.5 (20.3–25.0)	25.2 (23.3–28.5)	<0.001
Handgrip strength, % (*n* = 175)	83.7 (66.3–100.0)	90.7 (72.2–107.0)	79.7 (65.1–95.3)	<0.01
Waist circumference (cm)	95.0 (86.5–104.5)	84.0 (78.0–91.0)	100.0 (94.5–107.0)	<0.001
Hemoglobin, g/L	114 (104–121)	112 (102–121)	114 (105–122)	ns
Albumin, g/L	34.0 (31.0–37.0)	34.0 (30.0–37.0)	34.5 (31.0–37.0)	ns
Triglyceride, mmol/L	1.5 (1.2–2.2)	1.5 (1.1–2.0)	1.6 (1.2–2.2)	ns
Total cholesterol, mmol/L	4.3 (3.6–5.2)	4.8 (3.8–5.6)	4.3 (3.5–5.0)	<0.05
HDL-cholesterol, mmol/L	1.2 (1.0–1.6)	1.5 (1.2–1.8)	1.2 (1.0–1.4)	<0.001
LDL-cholesterol, mmol/L	2.7 (2.0–3.4)	2.9 (2.2–3.6)	2.6 (2.0–3.3)	<0.05
Calcium, mmol/L	2.3 (2.1–2.4)	2.3 (2.2–2.4)	2.3 (2.1–2.4)	ns
Phosphate, mmol/L	1.8 (1.5–2.1)	1.8 (1.5–2.3)	1.8 (1.5–2.0)	ns
iPTH, ng/L	274 (190–443)	264 (160–453)	292 (197–429)	ns
hsCRP, mg/L	1.5 (0.8–4.8)	1.0 (0.6–2.0)	2.2 (0.9–6.0)	<0.001
IL-6, pg./ml (*n* = 126)	4.0 (2.1–7.8)	2.3 (0.9–4.5)	5.3 (2.3–8.8)	<0.001
ABSI	0.9 (0.8–0.9)	0.8 (0.8–0.8)	0.9 (0.9–0.9)	<0.001
AIP	0.2 (−0.2–0.6)	0.0 (−0.3–0.3)	0.3 (−0.1–0.8)	<0.01
CAC Score, AU	133.9 (0.0–1,224.9)	2.0 (0.0–246.8)	329.3 (12.6–1,488.0)	<0.001
AVC score, AU	0.0 (0.0–0.0)	0.0 (0.0–0.0)	0.0 (0.0–45.5)	<0.001
Beta-blocker (*n* = 197)	128 (65.0%)	39 (60.0%)	89 (67.4%)	ns
Ca-blocker (*n* = 174)	91 (52.3%)	31 (55.4%)	60 (50.8%)	ns
ACEi/ARB (*n* = 72)	54 (75%)	19 (79%)	35 (73%)	ns
Statin user (*n* = 198)	84 (42.4%)	19 (29.2%)	65 (48.9%)	<0.01

Data are presented as median (IQR, interquartile range) for continuous measures, and n (%) for categorical measures.

AVC, aortic valve calcium; ABSI, a body shape index; AIP, atherogenic index of plasma; CI, conicity index; CVD, cardiovascular disease; SBP, systolic blood pressure; DBP, diastolic blood pressure; FRS, Framingham CVD risk score; PEW, protein-energy wasting; SGA, subjective global assessment; BMI, body mass index; %HGS, hand grip strength, converted to % of sex-matched healthy controls; HDL, high-density lipoprotein; LDL, low-density lipoprotein; iPTH, intact parathyroid hormone; hsCRP, high sensitivity C-reactive protein; IL-6, interleukin-6; AU, Agatston units; CAC, coronary artery calcium; ACEi/ARB, angiotensin-converting enzyme inhibitor/angiotensin II receptor blockers.

**Table 2:** Baseline clinical and biochemical characteristics of 203 CKD5 patients according to tertiles of a body shape index (ABSI).

	All	Low tertile	Mid + high tertile	Value of *p*
	*N* = 203	*N* = 67	*N* = 136	
ABSI	0.9 (0.8–0.9)	0.8 (0.8–0.8)	0.9 (0.9–0.9)	<0.001
Age, years	56.0 (43.5–66.8)	47.0 (32.0–58.0)	60.5 (49.0–67.9)	<0.001
Male sex, *n* (%)	138 (68.0%)	39 (58.2%)	99 (72.8%)	<0.05
Diabetes mellitus, *n* (%)	34 (17.1%)	6 (9.2%)	28 (20.9%)	<0.05
CVD, *n* (%)	43 (21.5%)	12 (18.5%)	31 (23.0%)	ns
FRS%	13.6 (5.6–27.2)	7.4 (3.0–16.2)	17.1 (7.9–29.2)	<0.001
Systolic BP, mmHg	143 (130–153)	149 (130–156)	140 (129–151)	ns
Diastolic BP, mmHg	86 (76–93)	86 (79–95)	85 (76–92)	ns
Malnutrition (SGA)	77 (38.9%)	28 (42.4%)	49 (37.1%)	ns
Height, cm	173 (165–180)	171 (163–180)	174 (167–180)	ns
Weight, kg	74.0 (65.0–84.3)	74.0 (61.0–84.1)	74.3 (67.0–84.4)	ns
BMI, kg/m^2^	24.6 (22.2–27.5)	23.5 (21.3–26.9)	24.7 (22.5–27.7)	ns
Handgrip strength, % (*n* = 175)	83.7 (66.3–100.0)	93.0 (79.1–107.0)	76.7 (63.0–95.3)	<0.001
Waist circumference (cm)	95.0 (86.5–104.5)	87.5 (79.0–94.0)	99.0 (93.0–107.0)	<0.001
Hemoglobin, g/L	114 (104–121)	114 (102–122)	114 (105–121)	ns
Albumin, g/L	34.0 (31.0–37.0)	34.0 (31.0–37.0)	35.0 (31.0–37.0)	ns
Triglyceride, mmol/L	1.5 (1.2–2.2)	1.5 (1.2–1.9)	1.5 (1.1–2.3)	ns
Total cholesterol, mmol/L	4.3 (3.6–5.2)	4.8 (3.9–5.4)	4.3 (3.5–5.0)	<0.01
HDL-cholesterol, mmol/L	1.2 (1.0–1.6)	1.5 (1.2–1.8)	1.2 (1.0–1.4)	<0.001
LDL-cholesterol, mmol/L	2.7 (2.0–3.4)	2.8 (2.2–3.6)	2.6 (1.9–3.3)	ns
Calcium, mmol/L	2.3 (2.1–2.4)	2.3 (2.2–2.4)	2.3 (2.1–2.4)	ns
Phosphate, mmol/L	1.8 (1.5–2.1)	1.8 (1.6–2.2)	1.8 (1.5–2.1)	ns
iPTH, ng/L	274 (190–443)	245 (141–375)	292 (203–450)	ns
hsCRP, mg/L	1.5 (0.8–4.8)	1.1 (0.6–1.9)	2.4 (0.9–6.8)	<0.001
IL-6, pg./ml (*n* = 126)	4.0 (2.1–7.8)	2.6 (0.5–4.5)	5.1 (2.3–8.8)	<0.001
ABSI	1.3 (1.3–1.4)	1.2 (1.2–1.3)	1.4 (1.3–1.4)	<0.001
AIP	0.2 (−0.2–0.6)	0.1 (−0.3–0.4)	0.3 (−0.1–0.8)	<0.01
CAC Score, AU	133.9 (0.0–1,224.9)	7.9 (0.0–244.2)	329.5 (6.6–1,624.7)	<0.001
AVC score, AU	0.0 (0.0–0.0)	0.0 (0.0–0.0)	0.0 (0.0–36.5)	<0.001
Beta-blocker (*n* = 197)	128 (65.0%)	39 (60.0%)	89 (67.4%)	ns
Ca-blocker (*n* = 174)	91 (52.3%)	32 (59.3%)	59 (49.2%)	ns
ACEi/ARB (*n* = 72)	54 (75%)	16 (80%)	38 (73%)	ns
Statin user (*n* = 198)	84 (42.4%)	20 (30.8%)	64 (48.1%)	<0.05

Data are presented as median (IQR, interquartile range) for continuous measures, and n (%) for categorical measures.

AVC, aortic valve calcium; ABSI, a body shape index; AIP, atherogenic index of plasma; CI, conicity index; CVD, cardiovascular disease; SBP, systolic blood pressure; DBP, diastolic blood pressure; FRS, Framingham CVD risk score; PEW, protein-energy wasting; SGA, subjective global assessment; BMI, body mass index; %HGS, hand grip strength, converted to % of sex-matched healthy controls; HDL, high-density lipoprotein; LDL, low-density lipoprotein; iPTH, intact parathyroid hormone; hsCRP, high sensitivity C-reactive protein; IL-6, interleukin-6; AU, Agatston units; CAC, coronary artery calcium; ACEi/ARB, angiotensin-converting enzyme inhibitor/angiotensin II receptor blockers.

Baseline clinical and biochemical characteristics for men and women, respectively, are shown in Supplementary Table 1. Males were taller, heavier, and had a longer waist circumference, and CI and ABSI were slightly higher in males while cholesterol was significantly higher in females.

Patients were divided into two groups according to the distribution of lower tertile vs. the middle and higher tertiles of CI (Table 1) and ABSI (Table 2), respectively. Table 1 shows that patients with middle and higher CI (indicating greater abdominal fat deposition), compared to those with the lowest CI tertile, tended to be older, more often male, and diabetic, had lower concentrations of total cholesterol and lower levels of HDL-C, whereas concentrations of TG did not differ between the two patient groups. BMI, FBMI, and LBMI were significantly higher in patients with higher CI, whereas HGS tended to be weaker in patients with high CI (Table 1). The patients with higher CI had significantly higher levels of inflammation markers, such as hsCRP and IL-6 and significantly higher AIP, CAC and AVC scores. There were no significant differences in the systolic and diastolic blood pressure or presence of CVD between the two patient groups.

Table 2 shows that the patients with higher ABSI were older and included more males and diabetic patients. The patients with higher ABSI, compared to lower ABSI tertile, had lower concentrations of total cholesterol and lower levels of HDL-C, whereas TG did not differ between the two patient groups. Moreover, BMI, FBMI, and LBMI were not significantly different between the two patient groups, whereas HGS tended to be lower in patients with higher ABSI (Table 2). The patients with higher ABSI had significantly higher concentrations of hsCRP and IL-6. Table 2 shows that the patients with higher ABSI had significantly higher AIP, CAC and AVC scores. There were no significant differences in the systolic and diastolic blood pressure or presence of CVD between the two patient groups.

Table 3 displays a Spearman rank correlation matrix of associations between adiposity indices CI and ABSI and cardiovascular indices FRS and AIP. Both CI and ABSI were significantly associated with CAC and AVC scores, FRS and AIP. Moreover, CI but not ABSI, was significantly correlated with AVC. As expected, CI showed strong correlation (rho = 0.92) with ABSI. BMI was associated with CI but did not correlate with ABSI.

**Table 3:** Spearman rank correlation matrix for seven variables representing anthropometric measurements and cardiovascular risk indices in 203 CKD5 patients.

	BMI	CI	ABSI	CAC score	AVC score	FRS%
CI	0.39^c^					
ABSI	0.04	0.92^c^				
CAC score	0.20^b^	0.35^c^	0.29^c^			
AVC score	0.21^b^	0.30^c^	0.22^b^	0.45^c^		
FRS%	0.27^c^	0.43^c^	0.37^c^	0.69^c^	0.50^c^	
AIP	0.30^c^	0.27^c^	0.16^a^	0.20^b^	0.13	0.28^c^

^a^*p* < 0.05, ^b^*p* < 0.01 and ^c^*p* < 0.001.

AVC, aortic valve calcium; ABSI, a body shape index; AIP, atherogenic index of plasma; CI, conicity index; FRS, Framingham CVD risk score; BMI, body mass index; CAC, coronary artery calcium.

Univariate correlations of CI and ABSI with inflammatory markers, nutritional and anthropometric indices as well as lipids are shown in Supplementary Table 2. CI and ABSI were positively and significantly correlated with hsCRP and IL-6. CI was negatively and weakly associated with SGA, whereas ABSI did not show a significant correlation with SGA. Both CI and ABSI were negatively correlated with HGS. Serum albumin was not correlated with CI and ABSI. Among lipid parameters, HDL-C was negatively associated with CI and ABSI, whereas TG was positively associated with CI but did not significantly correlate with ABSI. Total cholesterol did not show a significant correlation with CI and ABSI. Moreover, in a linear regression analysis in a model including FRS, hsCRP, CAC and SGA as shown in Table 4, we found that CI was independently associated with FRS, CAC and SGA. However, ABSI was independently associated *only* with FRS.

**Table 4:** Multivariate linear regression analysis of parameters related to conicity index (CI) and a-body shape index (ABSI) in CKD5 patients.

	CI (adj *r*^2^ = 0.18)		ABSI (adj *r*^2^ = 0.10)	
	*β*	*p*	*β*	*p*
FRS	0.31	<0.001	0.230	0.003
hsCRP	0.38	0.573	0.064	0.355
CAC score	0.12	0.036	0.144	0.057
SGA	−0.21	0.002	−0.135	0.052

CI, conicity index; ABSI, a body shape index; FRS, Framingham CVD risk score; hsCRP, high sensitivity C-reactive protein; CAC, coronary artery calcium; SGA, subjective global assessment.

### 3.1. Follow-up data for cardiovascular events

**Figure 1:**
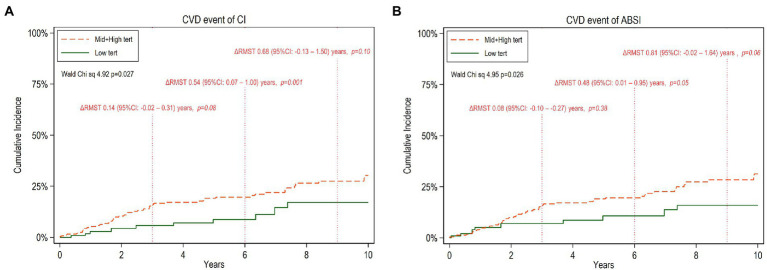
Cumulative incidence of cardiovascular disease (CVD) events during 10 years of follow-up among CKD5 patients with low tertiles versus high and middle tertiles of CI **(A)** and ABSI **(B)** respectively. The mean difference of restricted mean survival time, ΔRMST (95% confidence interval, 95%CI) between the two tertile groups at truncation time 3,6 and 9 years, respectively, is noted.

During the follow-up period of up to 10 years (median 5.7 years, IQR 2.4–9.8 years), the patients experienced 59 CVE. Compared to those free of CVE, the patients who faced CVE had a higher proportion of diabetics (33 vs. 10%; *p* < 0.001) and had higher hsCRP concentration (3.8 vs. 1.3 mg/l; *p* < 0.001), AIP (0.41 vs. 0.11; *p* < 0.001), CI (1.37 vs. 1.32; *p* < 0.001), ABSI (0.86 vs. 0.84; *p* = 0.005) and BMI (25.2 vs. 24.2 kg/m^2^; *p* < 0.05). However, there was no significant difference in nutritional status, as reflected by SGA, between the two groups. In multivariate age-weighted analysis, adjusted for gender, DM, CVD, and total cholesterol, the cumulative incidence curve showed that patients with middle and higher tertiles of CI and ABSI, respectively, had higher risk of CVE compared to those with lower CI (Wald test: 4.92, *p* = 0.027) and ABSI (Wald test: 4.95, *p* = 0.026) tertiles, respectively. For estimating the CV event-free time for low vs. middle and high tertiles of CI and ABSI, respectively, for the whole follow up period, or the mean event-free time during a prespecified period, we calculated multivariate ΔRMST representing the mean absolute difference of event-free time between the CI or ABSI tertile groups during the follow-up period. At 10 years of follow-up, the ΔRMST (Table 5), showing the difference between low vs. middle and high tertiles was 0.92 [95% CI, −0.03–1.87] years for CI and 1.12 [95% CI, 0.15–2.09] years for ABSI, indicating that the patients with the low tertiles had longer time free of CVE than the patients with the middle and high CI or ABSI tertiles, respectively. Moreover, in further analysis, Figures 1A,B show point estimates of RMST at 3, 6, and 9 years. As shown in Figures 1A,B, ΔRMST, i.e., the incremental benefit in event-free time of low tertile over high and middle tertiles of CI, was 0.14 years at 3 years, 0.54 years at 6 years, and 0.68 years at 9 years. All differences at 6 years but not at 3, 9, and 10 years were statistically significant. The corresponding incremental benefit (i.e., ΔRMST) of low tertile over high and middle tertiles of ABSI was 0.08, 0.48 and 0.81 years at 3, 6 and 9 years, respectively. Differences at 6 and 10 years but not at 3 years and 9 years were statistically significant.

**Table 5:** The age-weighted restricted mean survival time (RMST) analysis adjust for gender, DM, CVD, and total cholesterol.

	Cardiovascular events					
	CI			ABSI		
	Estimate (years)	[95% CI]	Value of *p*	Estimate (years)	[95% CI]	Value of *p*
ΔRMST at 10 years	0.92	[−0.03–1.87]	0.06	1.12	[0.15–2.09]	0.02
ΔRMST at 9 years	0.68	[−0.13–1.50]	0.10	0.81	[−0.02–1.64]	0.06
ΔRMST at 6 years	0.54	[0.07–1.00]	0.02	0.48	[0.01–0.95]	0.05
ΔRMST at 3 years	0.14	[−0.02–0.31]	0.08	0.08	[−0.10–0.27]	0.38

The RMST difference (ΔRMST) between low tertile versus high and middle tertiles of conicity index (CI) and a-body shape index (ABSI) regarding cardiovascular events during 10 years of follow-up among 203 CKD5 patients.

The same analyses were also performed separately for females and males (Supplementary Tables 3, 4; Supplementary Figures 1, 2). In men, low tertiles associated with a significant benefit in RMST compared to high and middle tertiles, but, for women, there was no clear RMST benefit, perhaps because the numbers are insufficient for analysis.

### 3.2. Follow-up data for survival analysis

During a follow-up period of up to 10 years, there were 69 deaths (34%). Compared to alive patients during the follow-up period, the group of patients who died had a higher prevalence of diabetics (29 vs. 11%; *p* < 0.001), CVD (37 vs. 14%; *p* < 0.001), higher levels of CRP (4.2 vs. 1.1 mg/l; *p* < 0.001), and higher AIP (0.35 vs. 0.12; *p* < 0.001), CI (1.37 vs. 1.32; *p* < 0.001) and ABSI (0.86 vs. 0.84; *p* < 0.01). There were no significant differences according to SGA and BMI between the patients who died and those who lived. Age-weighted survival analysis adjusted by gender, DM, CVD, and total cholesterol showed that the low CI tertile and low ABSI tertiles were associated with better survival than the middle and higher CI tertiles (Wald test = 5.24, *p* = 0.022) and middle and higher ABSI tertiles (Wald test = 5.19, *p* = 0.023), respectively.

**Figure 2:**
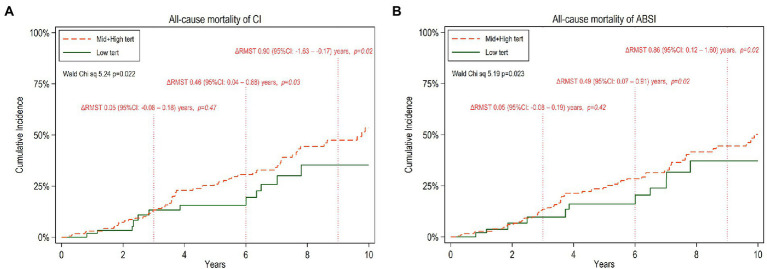
Cumulative incidence of all-cause mortality for 10 years follow-up of CKD5 patients with low versus high and middle tertiles of CI **(A)** and ABSI **(B)** respectively. The mean difference between the two tertile groups of restricted mean survival time truncated at 3, 6, and 9 years, ΔRMST with 95% confidence interval (95%CI), is noted.

For estimating all-cause mortality associated with low vs. middle and high tertiles of CI or ABSI, we applied RMST analysis and calculated ΔRMST. Figures 2A,B show point estimates of RMST at 3, 6 and 9 years for all-cause mortality. The ΔRMST (Table 6) shows that the difference in survival time for low tertile vs. high and middle tertiles of CI was 0.05, 0.46, 0.90 and 1.19 years at 3, 6, 9 and 10 years, respectively. The corresponding figures for low tertile vs. high and middle tertiles of ABSI was 0.05, 0.49, 0.86 and 1.09 years at 3, 6, 9 and 10 years, respectively. The incremental benefit of low CI and low ABSI at 6, 9 and 10 years was statistically significant while this was not the case at 3 years.

**Table 6:** The age-weighted restricted mean survival time (RMST) analysis adjusted for gender, DM, CVD, and total cholesterol.

	All-cause mortality					
	CI			ABSI		
	Estimate (years)	[95% CI]	Value of *p*	Estimate (years)	[95% CI]	Value of *p*
ΔRMST at 10 years	1.19	[0.32–2.06]	0.01	1.09	[0.20–1.98]	0.02
ΔRMST at 9 years	0.90	[−1.63–0.17]	0.02	0.86	[0.12–1.60]	0.02
ΔRMST at 6 years	0.46	[0.04–0.88]	0.03	0.49	[0.07–0.91]	0.02
ΔRMST at 3 years	0.05	[−0.08–0.18]	0.47	0.05	[−0.08–0.19]	0.42

The RMST difference (ΔRMST) between low tertile versus high and middle tertiles of conicity index (CI) and a-body shape index (ABSI) regarding all-cause mortality during 10 years of follow-up among 203 CKD5 patients.

## 4. Discussion

There is a consensus that adipose tissue location, and especially central obesity, rather than total adipose tissue mass, is of importance for the metabolic and inflammatory consequences of obesity in CKD, and WC has been shown to be a better predictor of outcomes than BMI in CKD patients (34). The CI and ABSI are two proposed indices to assess central obesity with the shared characteristic laying in the fact that they both are based on WC, with adjustment for height and weight. Accordingly, an elevated CI or ABSI indicate that WC is higher than expected for a given height and weight suggesting accumulation of adipose tissue around the abdominal region. In this study, we applied these anthropometric indices as estimates of central obesity in CKD stage 5 patients.

The current study demonstrated four major findings. First, higher CI and ABSI were associated with scores indicating increased cardiovascular risk, such as CAC and AVC scores, FRS and AIP. In addition, patients who had a history of CVD had higher values of CI and ABSI. Second, CI and ABSI were significantly associated with inflammatory status and negatively with HGS%. Third, CI and ABSI were associated with the risk of CVE during the follow-up period. Lastly, CI and ABSI were associated with all-cause mortality. These results show that the abdominal fat deposit indices, CI and ABSI, in CKD patients are significantly associated with several cardiovascular risk indices including inflammatory status and suggest that they could be of value for predicting cardiovascular outcomes and mortality among these patients.

It is generally acknowledged that CKD patients suffer from accelerated atherosclerosis and that CVD represents the leading cause of death in these patients (35). Adiposity is a known precursor of atherosclerosis and the increasing prevalence of obesity has implications for the risk of diabetes, CVD and also for development of CKD (6). Moreover, the prevalence of obesity has increased substantially among patients with CKD (36). Adiposity can be widely divided into general adiposity, which is usually assessed by BMI, and into central adiposity, that is reflected by WC. Central obesity is believed to be more pathogenic and more important as a predictor of cardiovascular metabolic disease compared to general obesity (37, 38) and may have a greater association with metabolic health risks. Indeed, the central obesity indices as CI and ABSI, which are calculated from WC, are associated with higher risk of CVD in the general population (23, 39, 40).

Like previous reports (21, 41), in the current study, CKD patients with higher CI or ABSI were older, more often male, had diabetes and had signs of dyslipidemia, other cardiovascular risk indices and inflammatory state. Moreover, our study showed that the patients with high CI tertiles had higher BMI, fat body mass and lean body mass, whereas the tertiles of ABSI did not show differences in BMI, fat body mass and lean body mass. Although nutritional status, as assessed by SGA, did not differ between the tertiles of CI and ABSI likely since both indices are related with adiposity and can mask malnutrition when assessed by SGA, handgrip strength (HGS) showed strong reverse associations with both CI and ABSI. In a previous study, Cordeiro et al. (21) reported that HD patients with an increased CI tended to be more malnourished and had weaker HGS. HGS is a useful tool for the systematic assessment of muscle strength related to nutritional status. Reduced HGS is a common finding among CKD patients and strongly associated with morbidity and mortality (42). Similar to our findings of reverse associations of HGS with ABSI and CI, Krakauer and Krakauer (43) reported that HGS was inversely associated with ABSI in the National Health and Nutrition Examination Survey (NHANES) 2011–2014 data including 9,803 adults in the United States population. This association may also sign for low muscle function, a finding that can occur concomitantly with higher adiposity (44).

Our results demonstrated that central obesity, as reflected by CI and ABSI, was positively associated with two measures of cardiac calcification, CAC as well as AVC. The association of CI with CAC and AVC was stronger than that of ABSI with CAC and AVC. This was supported by the findings of multivariate regression analysis that CI was independently associated with FRS and CAC and SGA in a model including FRS, hsCRP, CAC and SGA, whereas in an alternative analysis including the same variables ABSI was independently associated with FRS, but not with CAC and other variables (Table 4). The association of central obesity with risk of coronary atherosclerosis was supported by our findings that AIP correlated with CI and ABSI. However, these findings are not in accordance with a previous study showing that CI was not correlated with AIP in relatively lean maintenance hemodialysis patients (45). The AIP is a marker of atherogenicity, and it is considered as an independent predictor of rapid progression of coronary atherosclerosis (46). Therefore, the association of AIP with the central obesity indices in the current study may underline the relationship of central obesity with cardiovascular risk. Indeed, one of the interesting findings in this study is that FRS was strongly correlated with CI and ABSI. The relationship between obesity and cardiovascular diseases is well known and is predominantly related to the visceral accumulation of fat depots. Obesity, in particular visceral obesity, is a well-known risk factor of CVD and CKD patients are subjected to accelerated atherosclerosis and frequently suffer from vascular calcification.

Excessive accumulation of visceral fat is associated with inflammation and linked to atherosclerotic events (47). In CKD patients, abdominal fat has been reported to be associated with inflammation (21, 48). In agreement with another study in HD patients (49), we found that high CI and ABSI were correlated with increased concentrations of CRP and IL-6, suggesting that abdominal fat deposition could be a significant contributor to increased CRP production in HD patients. Altogether these findings are adding further support to the concept of abdominal obesity being a promotor for inflammation and a risk factor for CVD in CKD patients. This concept is supported by our findings that high CI and ABSI in our patients were associated with a higher risk for CVE and higher all-cause mortality risk. This is consistent with a previous study that showed a relationship between CI and total mortality in prevalent HD patients (21). Moreover, in the current study, using RMST, the patients with low CI or ABSI had incremental benefits of increased CV event-free time and prolonged survival over the patients with high CI or high ABSI. Notably, these benefits, which were small before 3 years, increased steadily and showed a substantial improvement during the 10 years follow-up period.

The strengths of this study include detailed phenotyping of patients using anthropometric, imaging and laboratory measurements with few missing values and no patient being lost to follow up. The study also has some limitations that should be considered when interpreting the results. Firstly, as in any observational study, causality cannot be inferred. Secondly, we performed age-weighted analysis and considered several potential confounding factors such as gender, DM, CVD, and total cholesterol use but acknowledge the existence of residual and unknown confounding, and that the relatively small number of CKD patients may not have provided enough statistical power. Secondly, inclusion of both dialyzed and non-dialyzed patients may limit the interpretation. Thirdly, we rely on anthropometric indices for estimation of abdominal fat based only on WC. Nevertheless, WC has been validated against assessment of fat mass by computed tomography in CKD patients (50), and associations of anthropometric indices with visceral fat and metabolic risk indicators are in general as strong as those obtained by magnetic resonance imaging for measuring adipose tissue stores (51). Finally, because body weight and anthropometrics can be influenced by the hydration state, fluid status may have influenced the anthropometric indices.

In conclusion, the present study shows that abdominal body fat indices, in particular CI and ABSI, associate with cardiovascular risk indices, poor CV outcome and inflammatory status in CKD5 patients. It indicates that central obesity is a factor of importance when predicting CV outcomes and suggests that it may represent a mortality risk factor. Further studies on a larger scale are needed to confirm these findings.

## Data availability statement

The raw data supporting the conclusions of this article will be made available by the authors, without undue reservation.

## Ethics statement

The studies involving human participants were reviewed and approved by The Ethics Committee of the Karolinska Institute (EPN) at the Karolinska University Hospital Huddinge, Stockholm, Sweden. The patients/participants provided their written informed consent to participate in this study.

## Author contributions

KR, MS, ZC, AQ, BL, and PS: conceptualization. KR, AQ, BL, OH, PB, and PS: data curation. OH, PB, TB, JR, CA, PS, AQ, and BL: investigation. BL, PS, AQ, and MS: project administration. BL and PS: supervision. MS, KR, BL, and AQ: writing—original draft. All authors contributed to the article and approved the submitted version.

## Funding

Baxter Novum is the result of a grant from Baxter Healthcare Corporation to Karolinska Institutet.

## Conflict of interest

The authors declare that the research was conducted in the absence of any commercial or financial relationships that could be construed as a potential conflict of interest.

## Publisher’s note

All claims expressed in this article are solely those of the authors and do not necessarily represent those of their affiliated organizations, or those of the publisher, the editors and the reviewers. Any product that may be evaluated in this article, or claim that may be made by its manufacturer, is not guaranteed or endorsed by the publisher.

## Supplementary material

The Supplementary material for this article can be found online at: https://www.frontiersin.org/articles/10.3389/fnut.2023.1035343/full#supplementary-material
