# Supplementary material for: Central obesity as assessed by conicity index and a-body shape index associates with cardiovascular risk factors and mortality in kidney failure patients
Source: Front Nutr. 2023 Mar 1;10:1035343. doi: 10.3389/fnut.2023.1035343 (PMC10016612; doi:10.3389/fnut.2023.1035343)
Supplement: Supplementary file 1 [file Data_Sheet_1.docx]

Supplementary Material

# Supplementary Tables

Table S1. Baseline clinical and biochemical characteristics in 203 CKD5 patients according to gender

|  | **Total** | **Female** | **Male** | **p-value** |
| --- | --- | --- | --- | --- |
|  | **N=203** | **N=65** | **N=138** |  |
| CI | 1.3 (1.3-1.4) | 1.3 (1.2-1.4) | 1.4 (1.3-1.4) | <0.001 |
| ABSI | 0.9 (0.8-0.9) | 0.8 (0.8-0.9) | 0.9 (0.8-0.9) | 0.006 |
| Age, years | 56.0 (43.5-66.8) | 59.0 (49.0-67.8) | 53.1 (42.0-65.3) | ns |
| Diabetes mellitus, n (%) | 34 (17.1%) | 12 (18.8%) | 22 (16.3%) | ns |
| CVD, n (%) | 43 (21.5%) | 13 (20.3%) | 30 (22.1%) | ns |
| FRS% | 13.6 (5.6-27.2) | 10.4 (5.5-19.1) | 14.8 (5.6-29.3) | ns |
| Systolic BP, mmHg | 143.0 (130.0-153.0) | 142.0 (129.0-152.0) | 143.0 (130.0-153.0) | ns |
| Diastolic BP, mmHg | 86.0 (76.0-93.0) | 84.0 (74.0-93.0) | 86.0 (77.0-93.0) | ns |
| Malnutrition (SGA) | 77 (38.9%) | 30 (47.6%) | 47 (34.8%) | ns |
| Height, cm | 173.0 (165.0-180.0) | 164.0 (159.0-170.0) | 176.7 (171.5-182.0) | <0.001 |
| Weight, kg | 74.0 (65.0-84.3) | 64.4 (56.3-73.2) | 79.0 (70.0-88.0) | <0.001 |
| BMI, kg/m^2 | 24.6 (22.2-27.5) | 24.2 (21.0-27.9) | 24.6 (22.8-27.4) | ns |
| Handgrip strength, % (n=175) | 83.7 (66.3-100.0) | 74.1 (63.0-96.3) | 86.0 (69.8-100.0) | ns |
| Waist circumference (cm) | 95.0 (86.5-104.5) | 89.0 (82.0-98.0) | 97.3 (91.0-106.0) | <0.001 |
| Hemoglobin, g/L | 114.0 (104.0-121.0) | 114.0 (102.0-121.0) | 114.0 (105.0-122.0) | ns |
| Albumin, g/L | 34.0 (31.0-37.0) | 32.0 (29.5-35.0) | 35.0 (32.0-38.0) | <0.001 |
| Triglyceride, mmol/L | 1.5 (1.2-2.2) | 1.6 (1.2-2.2) | 1.5 (1.1-2.2) | ns |
| Total cholesterol, mmol/L | 4.3 (3.6-5.2) | 5.1 (4.0-5.8) | 4.1 (3.5-4.9) | <0.001 |
| HDL-cholesterol, mmol/L | 1.2 (1.0-1.6) | 1.6 (1.2-1.9) | 1.1 (1.0-1.4) | <0.001 |
| LDL-cholesterol, mmol/L | 2.7 (2.0-3.4) | 3.2 (2.1-3.7) | 2.6 (2.0-3.2) | 0.008 |
| Calcium, mmol/L | 2.3 (2.1-2.4) | 2.3 (2.2-2.4) | 2.3 (2.1-2.4) | ns |
| Phosphate, mmol/L | 1.8 (1.5-2.1) | 1.7 (1.5-1.9) | 1.8 (1.5-2.2) | ns |
| iPTH, ng/L | 273.5 (190.3-443.0) | 249.9 (149.5-451.3) | 292.3 (207.2-434.0) | ns |
| hsCRP, mg/L | 1.5 (0.8-4.8) | 1.7 (0.8-5.7) | 1.5 (0.7-4.6) | ns |
| IL-6, pg/ml (n=126) | 4.0 (2.1-7.8) | 4.5 (2.3-8.1) | 3.7 (1.8-7.4) | ns |
| AIP | 0.2 (-0.2-0.6) | 0.1 (-0.3-0.4) | 0.2 (-0.2-0.7) | 0.046 |
| CAC Score, AU | 133.9 (0.0-1,224.9) | 133.9 (0.0-668.0) | 129.0 (0.0-1,375.9) | ns |
| AVC Score, AU | 0.0 (0.0-0.0) | 0.0 (0.0-0.0) | 0.0 (0.0-34.5) | 0.010 |
| Beta-blocker (n=197) | 128 (65.0%) | 39 (62.9%) | 89 (65.9%) | ns |
| Ca-blocker (n=174) | 91 (52.3%) | 27 (49.1%) | 64 (53.8%) | ns |
| ACEi/ARB (n=72) | 54 (75%) | 21 (78%) | 33 (73%) | ns |
| Statin user (n=198) | 84 (42.4%) | 28 (45.2%) | 56 (41.2%) | ns |

Data are presented as median (IQR, interquartile range) for continuous measures, and n (%) for categorical measures.

Abbreviations: CI, conicity index; ABSI, a body shape index; AVC, aortic valve calcium; AIP, atherogenic index of plasma; CVD, cardiovascular disease; SBP, systolic blood pressure; DBP, diastolic blood pressure; FRS, Framingham CVD risk score; PEW, protein-energy wasting; SGA, subjective global assessment; BMI, body mass index; %HGS, hand grip strength, converted to % of sex-matched healthy controls; HDL, high-density lipoprotein; LDL, low-density lipoprotein; iPTH, intact parathyroid hormone; hsCRP, high sensitivity C-reactive protein; IL-6, interleukin-6; AU, Agatston units; CAC, coronary artery calcium; ACEi/ARB, angiotensin-converting enzyme inhibitor/ angiotensin II receptor blockers.

Table S2. Univariate correlation analysis of parameters of CKD5 patients related to CI and ABSI.

|  | CI | | ABSI | |
| --- | --- | --- | --- | --- |
|  | rho | p | rho | p |
| hsCRP | 0.26 | <0.001 | 0.23 | <0.001 |
| Interleukin-6 | 0.31 | <0.001 | 0.28 | <0.01 |
| SGA | -0.19 | <0.01 | -0.13 | ns |
| Albumin | 0.01 | ns | 0.03 | ns |
| HGS % | -0.24 | <0.01 | -0.27 | <0.001 |
| Total cholesterol | -0.11 | ns | -0.09 | ns |
| Triglycerides | 0.17 | <0.05 | 0.09 | ns |
| HDL-cholesterol | -0.27 | <0.0001 | -0.19 | <0.01 |

Abbreviations: CI, conicity index; ABSI, a body shape index; hsCRP, high sensitivity C-reactive protein; SGA, subjective global assessment; HGS%, handgrip strength, converted to % of sex-matched healthy controls; HDL, high-density lipoprotein.

**Table S3.** The restricted mean survival time (RMST) and the RMST difference (ΔRMST) between low (T1) versus high and middle (T2 & T3) tertiles of conicity index (CI) and a-body shape index (ABSI) regarding cardiovascular events and all-cause mortality during 10 years of follow-up in 65 female CKD5 patients.

| **Female patients (n=65)** | Cardiovascular events | | All-cause mortality | |
| --- | --- | --- | --- | --- |
|  | CI | ABSI | CI | ABSI |
| RMST T1 (years) | 8.58  (7.27 - 9.90) | 8.69  (7.49 - 9.89) | 8.81  (7.73 - 9.88) | 8.60  (7.49 - 9.71) |
| RMST T2&T3 (years) | 7.85  (6.82 - 8.87) | 7.81  (6.76 - 8.86) | 7.05  (6.08 - 8.02) | 7.15  (6.18 - 8.13) |
| ΔRMST (years) | 0.74  (0.39 - 0.93) | 0.88  (0.72- 2.47) | 1.76  (0.31 - 3.21) | 1.45  (0.03 - 2.92) |
| ΔRMST P-value | ns | ns | = 0.02 | ns |

**Table S4.** The restricted mean survival time (RMST) and the RMST difference (ΔRMST) between low (T1) versus high and middle (T2& T3) tertiles of conicity index (CI) and a-body shape index (ABSI) regarding cardiovascular events and all-cause mortality during 10 years of follow-up among 138 male CKD5 patients.

| **Male patients** (n=138) | Cardiovascular events | | All-cause mortality | |
| --- | --- | --- | --- | --- |
|  | CI | ABSI | CI | ABSI |
| RMST T1 (years) | 9.27  (8.58 - 9.96) | 9.22  (8.48 - 9.97) | 9.23  (8.50 - 9.95) | 8.69  (7.81 - 9.58) |
| RMST T2&T3 (years) | 6.83  (6.01 - 7.65) | 6.85  (6.04 - 7.66) | 7.02  (6.29 - 7.76) | 7.27  (6.53 - 7.96) |
| ΔRMST (years) | 2.44  (1.37 - 3.52) | 2.37  (1.27- 3.47) | 2.21  (1.17 – 3.24) | 1.43  (0.28 - 2.58) |
| ΔRMST P-value | < 0.001 | < 0.001 | < 0.001 | = 0.02 |

# Supplementary Figures

Legends to figures:

**Figure S1.** Cumulative incidence of cardiovascular disease (CVD) events during 10 years of follow-up among male and female CKD5 patients with low tertile versus high and middle tertiles of CI (Figure S1a) and ABSI (Figure S1b) respectively.


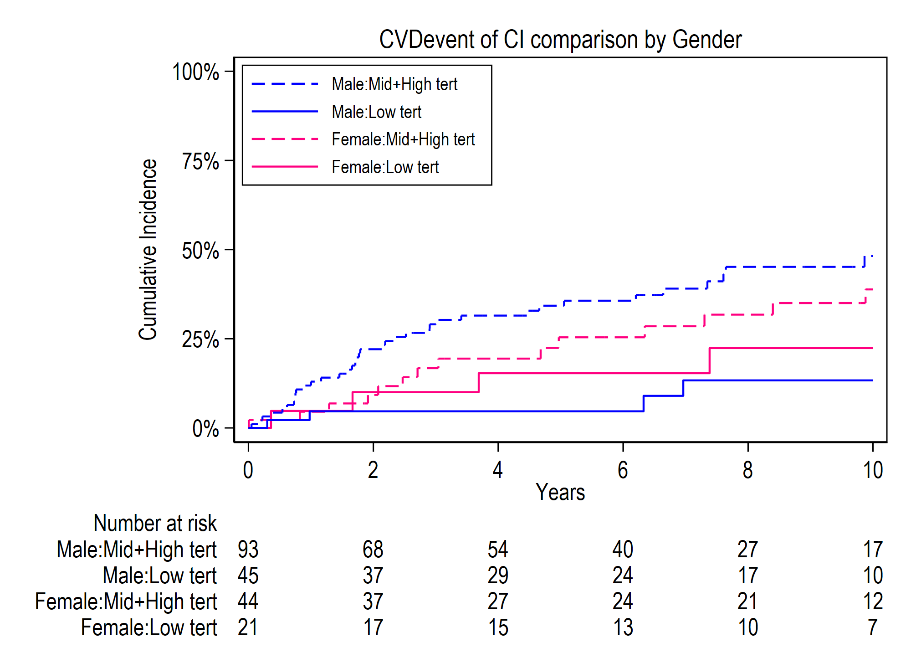
Fig. S1a Fig. S1b


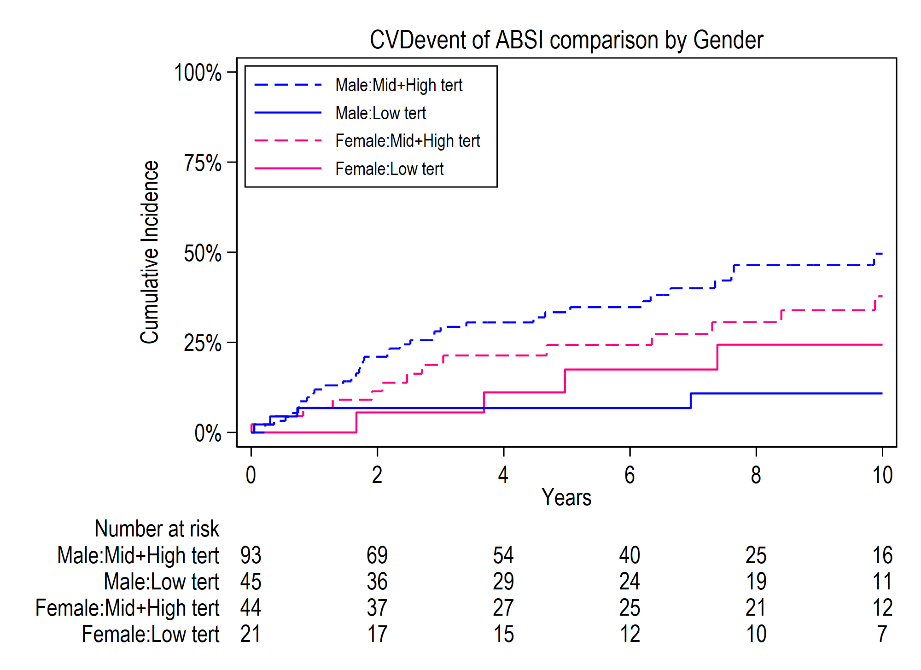


**Figure S2.** Cumulative incidence of all-cause mortality over 10 years follow-up of male and female CKD5 patients with low versus high and middle tertiles of CI (Figure S2a) and ABSI (Figure S2b) respectively.


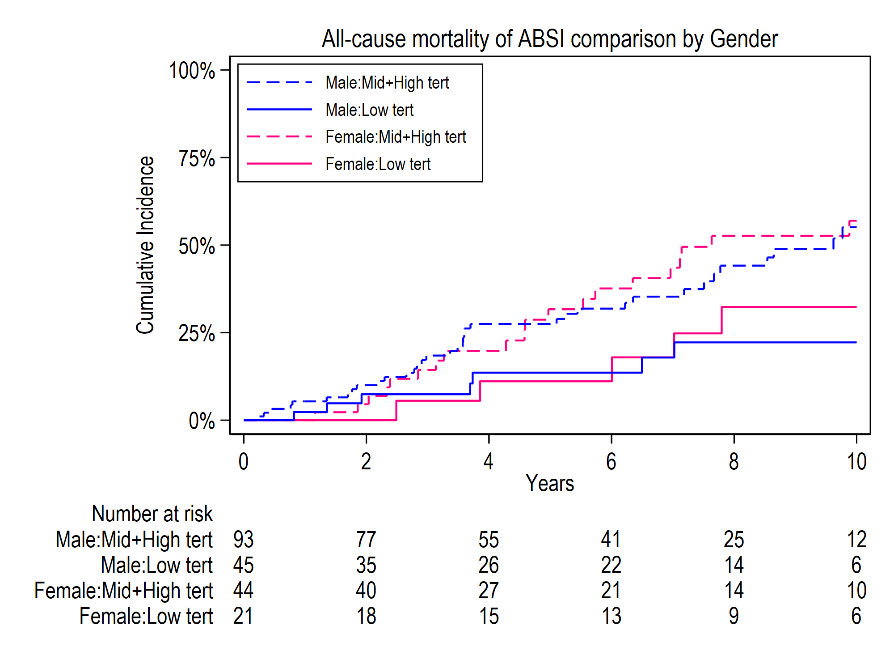

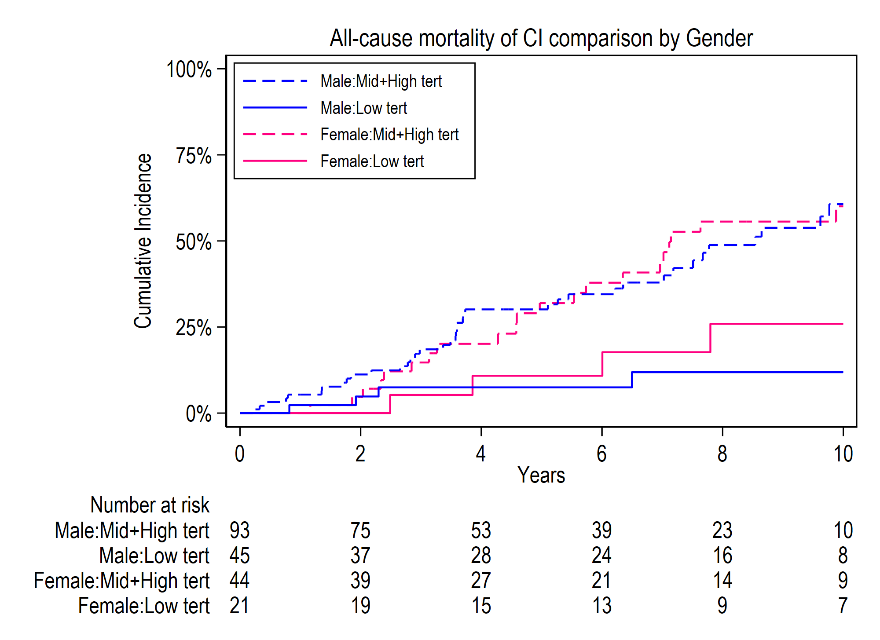
Fig. S2a Fig. S2b
